# Impacts on Sirtuin Function and Bioavailability of the Dietary Bioactive Compound Dihydrocoumarin

**DOI:** 10.1371/journal.pone.0149207

**Published:** 2016-02-16

**Authors:** Jennifer L. Jacobi, Bo Yang, Xu Li, Anna K. Menze, Sara M. Laurentz, Elsa M. Janle, Mario G. Ferruzzi, George P. McCabe, Clint Chapple, Ann L. Kirchmaier

**Affiliations:** 1 Department of Biochemistry, Purdue University, West Lafayette, Indiana, United States of America; 2 Purdue Center for Cancer Research, Purdue University, West Lafayette, Indiana, United States of America; 3 Department of Foods and Nutrition, Purdue University, West Lafayette, Indiana, United States of America; 4 Department of Food Science, Purdue University, West Lafayette, Indiana, United States of America; 5 Department of Statistics, Purdue University, West Lafayette, Indiana, United States of America; Texas A&M University, UNITED STATES

## Abstract

The plant secondary metabolite and common food additive dihydrocoumarin (DHC) is an inhibitor of the Sirtuin family of NAD^+^-dependent deacetylases. Sirtuins are key regulators of epigenetic processes that maintain silent chromatin in yeast and have been linked to gene expression, metabolism, apoptosis, tumorogenesis and age-related processes in multiple organisms, including humans. Here we report that exposure to the polyphenol DHC led to defects in several Sirtuin-regulated processes in budding yeast including the establishment and maintenance of Sir2p-dependent silencing by causing disassembly of silent chromatin, Hst1p-dependent repression of meiotic-specific genes during the mitotic cell cycle. As both transient and prolonged exposure to environmental and dietary factors have the potential to lead to heritable alterations in epigenetic states and to modulate additional Sirtuin-dependent phenotypes, we examined the bioavailability and digestive stability of DHC using an *in vivo* rat model and *in vitro* digestive simulator. Our analyses revealed that DHC was unstable during digestion and could be converted to melilotic acid (MA), which also caused epigenetic defects, albeit less efficiently. Upon ingestion, DHC was observed primarily in intestinal tissues, but did not accumulate over time and was readily cleared from the animals. MA displayed a wider tissue distribution and, in contrast to DHC, was also detected in the blood plasma, interstitial fluid, and urine, implying that the conversion of DHC to the less bioactive compound, MA, occurred efficiently *in vivo*.

## Introduction

Epigenetic processes controlling gene expression are influenced by both genetic and environmental factors. Humans are exposed to many compounds that act as modulators of epigenetic processes on a daily basis through their diet and environment. In recent years, many natural compounds found in botanicals, and often used as dietary factors and food additives, have been identified that affect the activity of enzymes critical for establishing and maintaining epigenetic states. Such enzymes range from those regulating histone acetylation or methylation to DNA methylation ([1] and references within).

Several plant polyphenolic compounds, including the flavanol quercetin and the stilbene piceatannol, stimulate the activity of the Sirtuins, an evolutionarily conserved family of NAD^+^-dependent deacetylases [2]. In contrast, other dietary bioactive compounds, including nicotinamide and the plant phenolic dihydrocoumarin (DHC) have been shown to inhibit Sir2 enzymes [3,4]. DHC is found naturally in many botanicals, including sweet clover (*Melilotus alba*, *Melilotus officinalis*), tonka bean (*Dipteryx odorata*), and deer tongue (*Carphephorus odoratissimus*), which have historically been used in teas, as flavorings for foods and in tobacco products [5–9]. Today, DHC, a FDA-approved dietary compound, is largely synthesized for use as a sweetener in foods including yogurt, ice cream, and soft drinks [10] as well as an aroma enhancer in cosmetics and perfumes [8]. The limited animal studies evaluating the safety and metabolism of DHC were performed prior to the discovery of Sirtuins and focused mainly on assessing toxicity of coumarins, with limited analyses of the tissue distribution of coumarin derivatives [11–14]. As DHC is a potential dietary modulator of Sirtuin-dependent processes, in this study, we investigated a range of effects of DHC on yeast Sirtuin-dependent phenotypes as well as conducted detailed pharmacokinetics in Sprague Dawley rats of DHC’s absorption, tissue distribution, metabolism and excretion after ingestion.

In *Saccharomyces cerevisiae*, the founding member of the Sirtuin family, Sir2p, is required for silent chromatin at the silent mating-type loci, telomeres and rDNA locus. Sir2p together with Sir1p, Sir3p, and Sir4p form the structural components of silent chromatin that spread across chromosomal loci and heritably inactivate gene expression through a mechanism that is driven by the histone deacetylase activity of Sir2p [15]. Misregulation of Sir2p causes silencing defects leading to the inability to mate [15,16], loss of telomere integrity [17], as well as rDNA instability [18] and influences the initiation of DNA replication [19]. Other members of the yeast Sirtuins are involved in mitotic repression of mid sporulation genes and regulation of origin firing (Hst1p) [20,21], in regulation of telomeric silencing and DNA damage responses through the deacetylation of H3 K56ac (Hst3p, Hst4p) [22,23], in modulation of telomeric and rDNA silencing (Hst2p) [24] and mitotic chromosome condensation through deacetylation of H4 K16 (Hst2p) [25].

In mammals, Sirtuins participate in a wide variety of processes ranging from epigenetic processes, transcription, chromosome stability and DNA repair, to metabolic homeostasis (including glucose metabolism, insulin secretion, lipid mobilization and fatty acid oxidation, cellular energy status and stress resistance), cardiovascular biology, the circadian clock, inflammation, apoptosis and senescence. Through their misregulation, Sirtuins have been associated with aging and age-related diseases such as neurodegenerative disorders, metabolic syndromes and cardiovascular disease as well as both the promotion and suppression of tumorogenesis [2,26–43]([44,45] and references within).

Humans express seven Sirtuins, SIRT1-7, several of which can also exhibit protein NAD^+^-dependent mono-ADP-ribosylase activity (e.g. SIRT4 and SIRT6) or demyristoylization and depalmitoylation activity (SIRT6) [40,46–49]. The closest homolog to yeast Sir2p and the best-characterized of the mammalian Sirtuins, SIRT1, deacetylates numerous targets including both histones and non-histone proteins, leading to transcriptional repression or altered protein activity to influence many of the processes noted above (see also [40–43] and references within). SIRT2 is both cytoplasmic and nuclear, deacetylates tubulin and histones, and is involved in regulating cell cycle progression [50–52]. SIRT3-5 are mitochondrial proteins involved in regulating respiration, energy production and entry of ammonia into the urea cycle [53–56]. SIRT6 deacetylates both histones [57–60] and non-histone proteins (e.g. [48]), and has been linked to numerous processes including DNA repair [48,61–63], transcriptional inactivation [64] and lipid and glucose metabolism (see [44] and references within) as well as aging-associated pathologies and lifespan [63,65]. The deacetylase SIRT7 regulates H3 K18ac-, ELK4- and MYC-dependent gene expression, as well as rRNA and tRNA synthesis [45,66–70]. Thus, modulation of the activity of Sirtuins through exposure to environmental and dietary factors has the potential to impact numerous cellular processes and affect the overall physiology and health of an organism.

In this study, we sought to understand the impact of the dietary bioactive compound DHC by determining the effects of DHC on chromatin composition and epigenetic gene regulation as well as its bioaccessibility and pharmacokinetics. Our studies have revealed that exposure to DHC causes disassembly of silent chromatin and leads to defects in both establishing and maintaining heritably silent transcriptional states as well as de-repression of meiotic-specific genes. We determined the fate of DHC upon ingestion using an *in vitro* model to simulate digestion events prior to absorption plus an *in vivo* rat model to assess bioavailability. Digestion simulations indicate DHC begins to be converted to melilotic acid (MA) during the gastric phase of digestion. Pharmacokinetics demonstrated DHC and its major metabolite MA exhibited partially overlapping tissue distributions, and were rapidly cleared from the body.

## Materials and Methods

### Yeast strains and plasmids

Yeast strains used in this study are listed in S1 Table A in S1 File. Yeast strains were generated using standard techniques, which include transformation, plasmid shuffling, homologous recombination, and one-step gene conversion [71]. Plasmids are listed in Table B in S1 File, and those expressing histone mutants were created using site-directed mutagenesis, according to the Quick Change Site-Directed Mutagenesis Kit protocol (Stratagene, La Jolla, CA).

### Colony color assays

Colony color assays were performed as described previously [72,73]. Logarithmically growing yeast that contain *ADE2* between the ***E*** and ***I*** silencers at *HMR* were plated on rich (YPD) media containing varying concentrations of DHC and/or MA. Plates were incubated at 30°C for 2 days and stored at 4°C for three days to develop the color. Images were taken using a Leica MZ125 microscope and SPOT 4.1.1 imaging software [72]. In this assay, red colonies indicate that *ADE2* at *HMR* is silenced, white colonies indicate that *ADE2* is expressed, pink colonies indicate a defect in maintaining or inheriting silencing of *ADE2* (defect in the stability of silent chromatin), and sectored colonies signify a defect in establishing silencing (defect in forming silent chromatin *de novo*). In these experiments, media was autoclaved, cooled to 50–55°C in a water bath, then DHC, MA or DHC plus MA added to media just prior to pouring plates. Plates were used within ~24 hr.

### *TRP1* reporter assays

Ten-fold serial dilutions of yeast containing *TRP1* integrated between the ***E*** and ***I*** silencers at *HMR* were plated onto minimal (YM) media lacking tryptophan or synthetic complete media containing varying concentrations of DHC and incubated for two days at 30°C. Images were taken using a Leica MZ125 microscope and SPOT 4.1.1 imaging software. In these experiments, media was autoclaved, cooled to 50–55°C in a water bath, then DHC was added to media just prior to pouring plates. Plates were used within ~24 hr.

### RNA analyses

Total RNA was isolated from strains grown logarithmically for ~3 hr in YPD media containing varying concentrations of DHC, which was added immediately prior to inoculating cultures, and cDNA was generated for transcript analysis of ***a1*** from *HMR* or *SCR1* by quantitative real time PCR, performed on an ABI Prism 7000, as described previously [74,75]. Experiments were performed in triplicate and the average and standard deviation (SD) was calculated for each sample, except where noted. Oligonucleotides used for this analysis are listed in Table C in S1 File. Statistical analyses were performed using the Wilcoxon rank sum test with MSTAT v.2.6 (http://mcardle.oncology.wisc.edu/mstat).

### Chromatin immunoprecipitation

Strains were grown logarithmically for 3 hr in the presence or absence of 200 μM DHC prior to conducting Chromatin immunoprecipitation (ChIP) experiments. ChIP experiments were performed and analyzed by real time PCR on an ABI Prism 7000 as described previously [75]. Oligonucleotides used for ChIP experiments are listed in Table C in S1 File.

### Immunoblot analyses

5 OD of mid-log phase cells grown in the presence or absence of 200 μM DHC for 4 hr were pelleted and resuspended in SDS-PAGE loading buffer (100 mM Tris-HCl, pH 6.8, 10% SDS, 10% glycerol, 0.04% bromophenol blue, 5% ß-mercaptoethanol) with protease inhibitors (0.1 mM TPCK, 1 mM PMSF, 5 μg/mL chymostatin, 2 μg/mL pepstatin A, 1 mM benzamidine). Samples were boiled for 1 min, glass beads were added, and samples vortexed to lyse cells. 0.33 OD cell equivalents were loaded onto and electrophoresed on a 15% SDS-PAGE gel. Proteins were transferred to PVDF membranes (Bio-Rad) in transfer buffer (25 mM Tris, 1.44% glycine, pH 8.3, 30% methanol). Membrane were blocked with TBS (20 mM Tris, pH 7.6, 0.8% NaCl) with 7% milk for 2 hr at room temperature and then incubated with anti-H3 K56ac antibody (Upstate, Cat. # 07–677; 1:10,000) over night at room temperature. Membranes were washed 2x with TBS + 0.1% Tween 20 for 5 min each, then washed 2x with TBS for 10 min. After washing, membranes were incubated with horseradish peroxidase-linked donkey anti-rabbit IgG, F(ab)'2 fragment (Amersham, Cat. #NA9340V; 1:20,000) for 1 hr at room temperature, then washed as above and visualized using an ECL-plus kit (Amersham) and Hyperfilm ECL (Amersham). Blots were stripped with 0.2 M NaOH at room temperature and reprobed with anti-histone H3 antibodies (Abcam Cat. # ab1791; 1:30,000) as above.

### α Factor confrontation assays

α-factor confrontation assays were performed by growing *bar1* yeast logarithmically, then adding 1 μg/ml α-factor in the presence or absence of varying concentrations of DHC at 30°C. Aliquots of cells were harvested at 3, 4, and 5 hr after the addition of α-factor and DHC. Cells were, sonicated briefly, pelleted by centrifugation and resuspended in 70% ethanol. Cell morphologies were scored by light microscopy. Schmoos and single cells were counted as being in G1 phase, small budded and “dumbbell” shaped yeast were scored as being in S phase, and large budded yeast were considered to be in G2/M phase.

### *In vitro* digestion

A two phase *in vitro* digestion assay using porcine digestive enzymes, similar to that developed by Garrett et al., was used to mimic the human digestive tract [76]. The dose equivalent for a 70 kg human was calculated by dividing the rat dose administered in this study (see In vivo bioavailability methods below) (100 mg/kg) by the standard FDA dose conversion factor (6.2) from rat to humans [77]. This dose was adjusted for use in a 50 ml simulated digestive system based on an ~2 L dilution in the human digestive tract. Emulsions of DHC or MA plus Tween 20 and water were generated by sonication for ~30 sec. This emulsion was added to 27.5 ml 0.9% saline for a final volume of 30 ml, and the pH of the mixture was recorded. The simulated gastric phase of the digestive tract was initiated by adding 3 ml of a pepsin solution [40 mg/ml pepsin (porcine stomach mucosa; Sigma-Aldrich) in 0.1 M HCl]. The pH was adjusted to <3.0, and samples were incubated in a dark 37°C shaking water bath for one hr. The gastric digesta was then neutralized by the addition of 3 ml of 100 mM NaHCO_3_. Simulation of the small intestinal phase of digestion was initiated by adding 4.5 ml of bile solution [24 mg/ml bile extract (porcine; Sigma-Aldrich) in 100 mM NaHCO_3_] and 4.5 ml of pancreatin-lipase solution [4 mg/ml pancreatin (porcine; Sigma-Aldrich) and 2 mg/ml lipase (Type II; porcine; Sigma-Aldrich) in 100 mM NaHCO_3_]. The pH of the mixture was adjusted to 6.5 by the addition of 100 mM NaHCO_3_. Saline solution was then added to bring the volume of each small intestinal phase simulation sample to 50 ml. Samples were then incubated in a dark shaking water bath at 37°C for two hrs. Immediately following the incubation, aliquots (digesta) were taken for analysis and stored at -80°C. The remainder of the samples was centrifuged at 10,000 x g for one hr to separate the aqueous micellar fraction from the non-digestible insoluble fraction. Aliquots of the bioaccessible aqueous fractions were stored at -80°C for later analysis.

### *In vivo* bioavailability

Animal protocols were approved by the Purdue Animal Care and Use Committee (PACUC). Fourteen Sprague Dawley rats were obtained from Harlan (Indianapolis, IN). Rats were fed a polyphenol free diet (AIN-93M, Dyets Inc., PN-101591, Bethlehem, PA) and had access to deionized water ad libitum for five days following arrival. Animals were kept on a 12 hr light/dark cycle in a climate-controlled facility. Five days after arrival, rats were anesthetized with 5% isoflurane (Baxter Healthcare Corporation, Deerfield, IL). Anesthesia was maintained with 3% isoflurane and a short femoral catheter (CX-2021S, BASi, West Lafayette, IN) was implanted into the femoral vein, and an ultrafiltrate probe (UF-3-12, BASi, West Lafayette, IN) was implanted subcutaneously along the dorsal midline. Buprenex (0.03 mg/ml, Reckitt Benskiser Healthcare, Hull, England) 0.1 ml/100 g of body mass was given subcutaneously as an analgesic prior to animal regaining sterna recumbence. Automatic blood and interstitial fluid draws was performed using a Culex automated sampling system (BASi, West Lafayette, IN), which infused 10 μl heparinized saline every 10 min to maintain catheter patency. Rats were given 100 mg/kg body mass dose of DHC in a 1 ml emulsion of DHC, water and Tween 20 by gavage (see in vitro digestion methods above). Food was restricted 8 hr prior to gavage and offered 2 hr post-gavage. The Culex^TM^ was used to automatically draw 250 μl of blood from the femoral catheter into heparinized tubes. Blood was centrifuged at 9,500 x g for 10 min at 4°C in a VWR Micro 18R centrifuge and 100 μl of resulting plasma was split into duplicate vials and 12.5 μl acidified saline (1% ascorbic acid wt/wt) was added to each vial. Interstitial fluid was collected continuously using a peristaltic pump (P721 Instech Laboratories, Plymouth Meeting, PA). Collection vials were changed every hour for 12 hr post-gavage to allow analysis at 1 hr time points. Vials containing blood plasma and interstitial fluid were purged with N_2_ and stored at -80°C until analysis. A second gavage was given two days after the first gavage and animals were sacrificed 1 hr or 6 hr post gavage. Rats were euthanized using the standard carbon dioxide rodent procedure (National Institute of Health, Office of Animal Care and Use, (HTTP://OACU.OD.NIH.GOV/ARAC/DOCUMENTS/RODENT_EUTHANASIA_ADULT.PDF). Blood was drawn immediately after euthanasia from the abdominal aorta, placed in heparinized tubes, centrifuged and stored as described above. The vascular system was flushed with 240 ml of cold saline to remove blood from the organs. Tissues were harvested, flash frozen in liquid nitrogen, and stored at -80°C.

### Tissue and plasma analyses

Secondary metabolites were extracted from tissues using an ethyl acetate extraction procedure [78]. Tissues or plasma were thawed in 3 ml ethyl acetate plus 0.01% 2,6-Di *tert*-butyl-4-methyl phenol (BHT) (Sigma-Aldrich), sonicated and subjected to centrifugation for 10 min at 1,015 x g. The organic layer was removed and the extraction was repeated without sonication twice more and pooled with other extracts. Samples were dried using a vacufuge, and the metabolites were redissolved in 50% methanol. Ten μilliliters of the redissolved sample or ultrafiltrate was analyzed by HPLC using a Shim-Pack XR-ODS column (3.0 mm x 75 mm) 0.33 μm (Shimadzu) and a gradient of 10 to 100% acetonitrile in 0.1% formic acid at a flow rate of 0.7 ml/min. Quantification was performed by determining the area under the curve and comparing to calibration curves constructed with multi-level concentrations of known standards. Interstitial fluid was analyzed directly on the HPLC without extraction.

### Data analyses

A mixed effects and repeated measures model [79] was used to determine the relationship among the concentrations of DHC or MA present and the time point at which urine, plasma, and interstitial fluid was collected. In these analyses, the rat was treated as the random effect to take the variation of each rat into account [79]. To determine the rate of decrease of MA in plasma, time was modeled as a quantitative variable and a regression line was fit to the data. Multiple comparisons of each compound between 1 and 6 hr for tissues or GI organ contents were performed using t-statistics that assume unequal variances. A mixed effects model in which the rat was treated as the random effect was used to test for differences in DHC/MA in tissues or GI organ contents. Mammalian data were analyzed using SAS statistical software (SAS Institute Inc, v 9.2), or MSTAT for Wilcoxon rank sum analyses of compounds in the GI tract.

## Results

### DHC disrupts silencing at *HMR*

**Fig 1:**
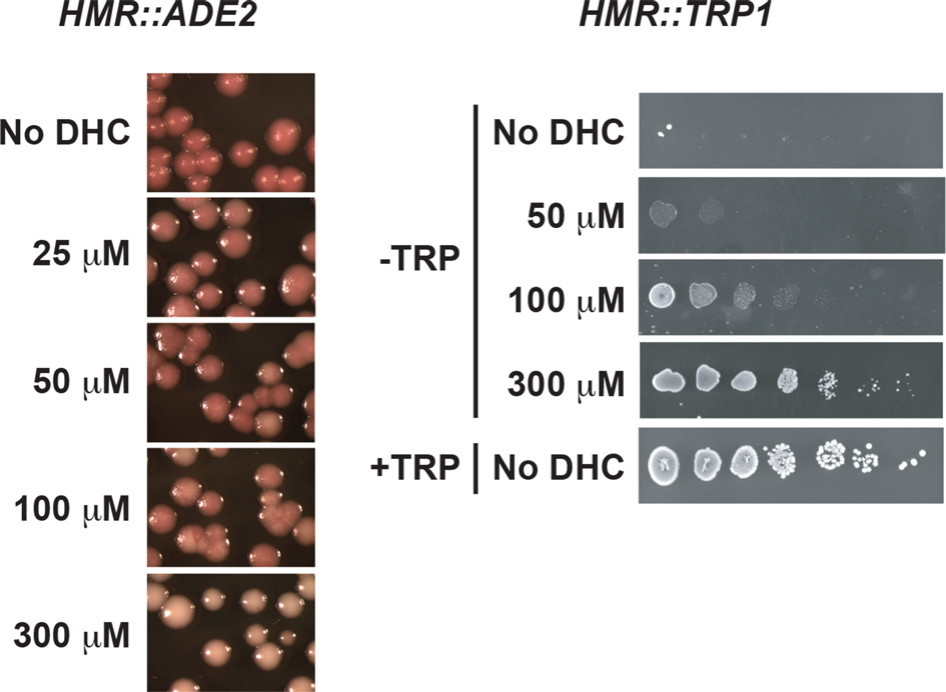
DHC inhibits silencing of reporter genes at *HMR*. In reporter strains, ***a1*** and ***a2*** at *HMR* were replaced by genes encoding A. *ADE2* or B. *TRP1*. Logarithmically growing yeast were plated on rich (YPD) media (**a**) or minimal (YM) media (**b**) containing or lacking tryptophan plus the indicated amount of DHC and analyzed as outlined in Materials and methods.

**Fig 2:**
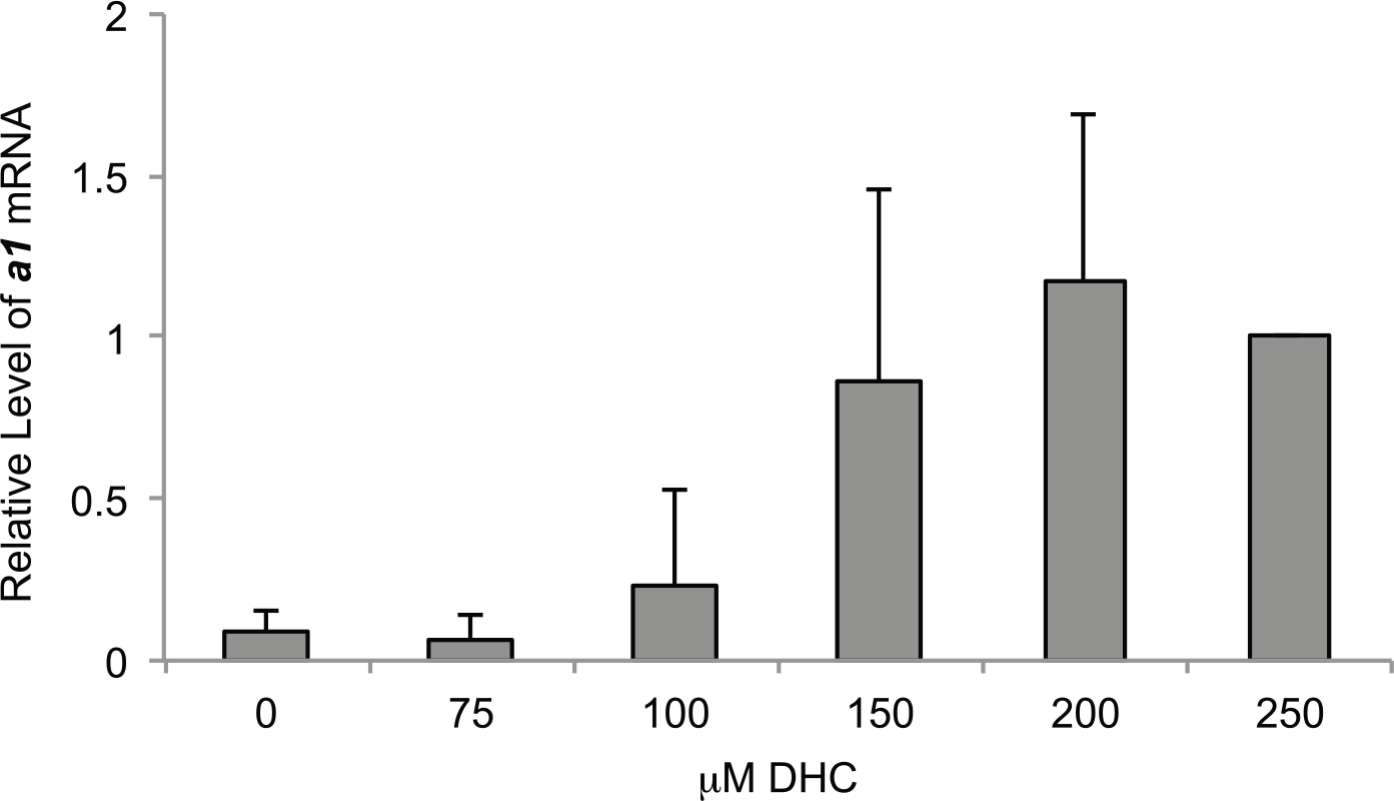
DHC inhibits silencing of *a1* at *HMR*. Transcript levels of ***a1*** relative to *SCR1*, an internal control, were measured by qRT-PCR in logarithmically growing cells in the presence or absence of DHC. The levels of transcripts present in samples treated with 250 μM DHC was set to 1. Data was calculated as: 2[(***a1***CT − *SCR1*CT)DHC − (***a1***CT − *SCR1*CT)No DHC] (AVG ± SD, n = 5).

Previously, DHC had been identified in screens for compounds that inhibit Sirtuins [7,80], but a detailed analysis of the effects of DHC on epigenetic states had not been conducted. To investigate how DHC impacted Sir2p-dependent epigenetic processes, we monitored mating-type silencing in yeast in which ***a1-a2*** at the silent mating-type locus *HMR* had been replaced with *ADE2* or *TRP1*. To identify whether DHC affected the establishment, maintenance or inheritance of epigenetic states, silencing at *HMR*::*ADE2* was evaluated by monitoring the colors of colonies of yeast grown in rich media containing or lacking DHC (See Materials and methods). Defects in maintaining or inheriting Sir2p-dependent silent chromatin were observed in the presence of 25 μM DHC (pink colonies) relative to the absence of DHC (red colonies), defects in establishing silencing were also observed at 50 and 100 μM DHC (sectored colonies) and silencing was completely disrupted in 300 μM DHC (white colonies) (Fig 1A). Similarly, when monitoring growth in the absence of tryptophan, silencing at *HMR*::*TRP1* in 50 μM DHC was reduced to ≤ 1% of that observed in the absence of DHC; these defects were more severe in 300 μM DHC (≤ 0.00001%) (Fig 1B). Consistent with DHC disrupting silencing in a gene-independent manner, transcript levels of ***a1*** from a native *HMR* locus were also elevated with increasing concentrations of DHC and ***a1*** became fully derepressed in 150 μM DHC (Fig 2). Together, these observations indicate that exposure to DHC both compromised the stability of silent chromatin and negatively influenced the probability of establishing the silenced state, and prompted us to investigate the nature of DHC-dependent defects in silent chromatin.

### DHC prevents Sir propagation

**Fig 3:**
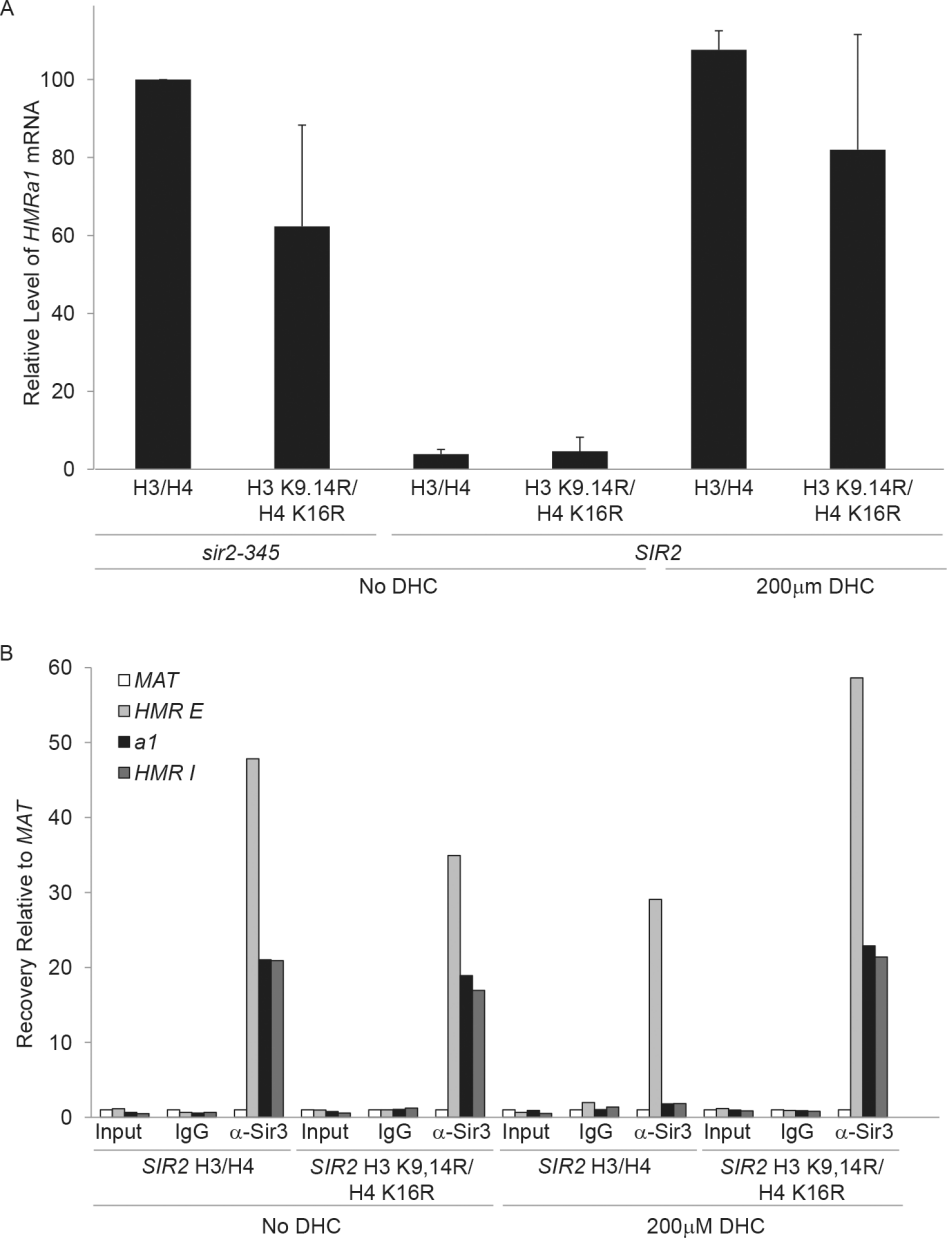
DHC prevents Sir spreading and mimics the catalytically inactive *sir2-345* mutant. **a** Transcriptional analysis by qRT-PCR of ***a1*** at *HMR* relative to an internal control (*SCR1*) was determined for strains with the indicated genotypes grown in rich (YPD) media in the absence or presence of 200 μM DHC, and compared to that observed in *sir2-345* mutants expressing wild-type H3 and H4, which was set to 100% (AVG ± SD, n = 3). Data was calculated as: 2[(***a1***CT − *SCR1*CT)Indicated strain − (***a1***CT − *SCR1*CT)*sir2-345* H3/H4]. **b** Sir3p binding at *MAT*, *HMR E* silencer, ***a1***, and *I* silencer in the absence or presence of 200 μM DHC was monitored by ChIP using IgG or anti-Sir3p antibodies and qRT-PCR in *SIR2* and *sir2-345* strains expressing wild-type or hypoacetylated H3/H4. Efficiency of co-precipitation of each locus is expressed relative to *MAT* and was calculated as: Locus IP/*MAT* IP = 2[(*MAT*CT − LocusCT); See also S2 Fig.

**Fig 4:**
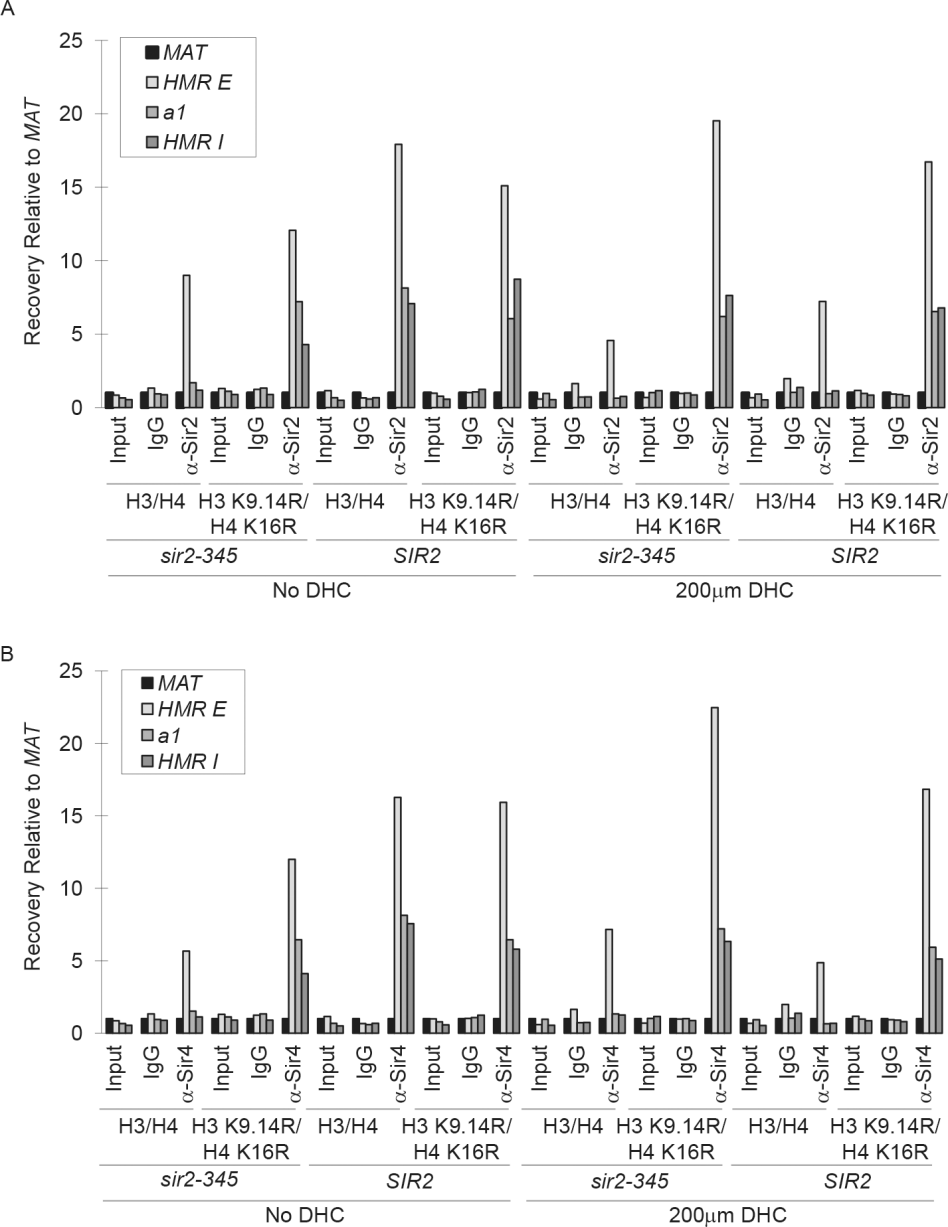
DHC prevents Sir spreading. **a** Sir2p and **b** Sir4p binding at *MAT*, *HMR-E*, ***a1***, and *HMR*-*I* in the presence or absence of DHC in *SIR2* or *sir2-345* strains expressing wild-type or hypoacetylated H3/H4 mutants were monitored by ChIP using IgG, anti-Sir2p and anti-Sir4p antibodies and qRT-PCR. The efficiency of co-precipitation of each locus is expressed relative to *MAT* and was calculated as described in Fig 3B; See also S2 Fig.

Silent chromatin formation at *HMR* requires the initial recruitment of Sir proteins to the *HMR-E* silencer and the subsequent propagation of Sirs throughout the locus as well as the catalytic activity of Sir2p [15,81–84] and references within). To gain mechanistic insight into how DHC affects the structure of silent chromatin, we monitored the association of Sir proteins at *HMR-E*, ***a1***, and *HMR-I* in *SIR2* cells expressing wild-type histones H3/H4 or H3/H4 lysine to arginine mutants that mimicked the hypoacetylated state in the presence or absence of DHC by ChIP (Fig 3B, Fig 4 and S2 Fig). In untreated cells expressing wild-type histones, Sir3p bound to *HMR-E* and spread throughout the *HMR* locus (Fig 3B) and ***a1*** was not expressed (Fig 3A; see also [85]). In contrast, in the presence of DHC, Sir3p was recruited to *HMR*-*E*, but failed to spread to ***a1*** and *HMR-I* and ***a1*** became derepressed (Fig 3A and 3B, and S2 Fig). This DHC-dependent defect in Sir association is similar to that observed in catalytically inactive *sir2-345* mutants in which sir2-345p, Sir3p and Sir4p are recruited to *HMR-E*, but do not spread throughout *HMR* or silence gene expression as sir2-345p cannot deacetylate histones [85,86]. In *SIR2* cells expressing H3 K9,14R H4 K16R hypoacetylation mutants, Sir3p spread across ***a1*** and *HMR*-*I* at *HMR* in both the absence and presence of DHC. However, in the presence of DHC, ***a1*** became derepressed indicating that silent chromatin had been disrupted (Fig 3A). Sir2p and Sir4p localization to *HMR* under these conditions was similar to that of Sir3p (Fig 4 and S2 Fig; see also [85]). This pattern of Sir association is analogous to our previous observations for *sir2-345* mutants in which expression of hypoacetylated histone mutants bypasses the requirement for the catalytic activity of Sir2p for Sir propagation, but not for silencing [85]. Together, these results are consistent with DHC having disrupted silencing by inhibiting the catalytic activity of Sir2p rather than by preventing interactions between Sir2p and silencer-binding proteins required for Sir recruitment to *HMR-E*, Sir-Sir or Sirs and hypoacetylated histones *per se* (see also [7,87]).

### DHC has varying impacts on Hst1p and Hst3p/Hst4p-dependent phenotypes

**Fig 5:**
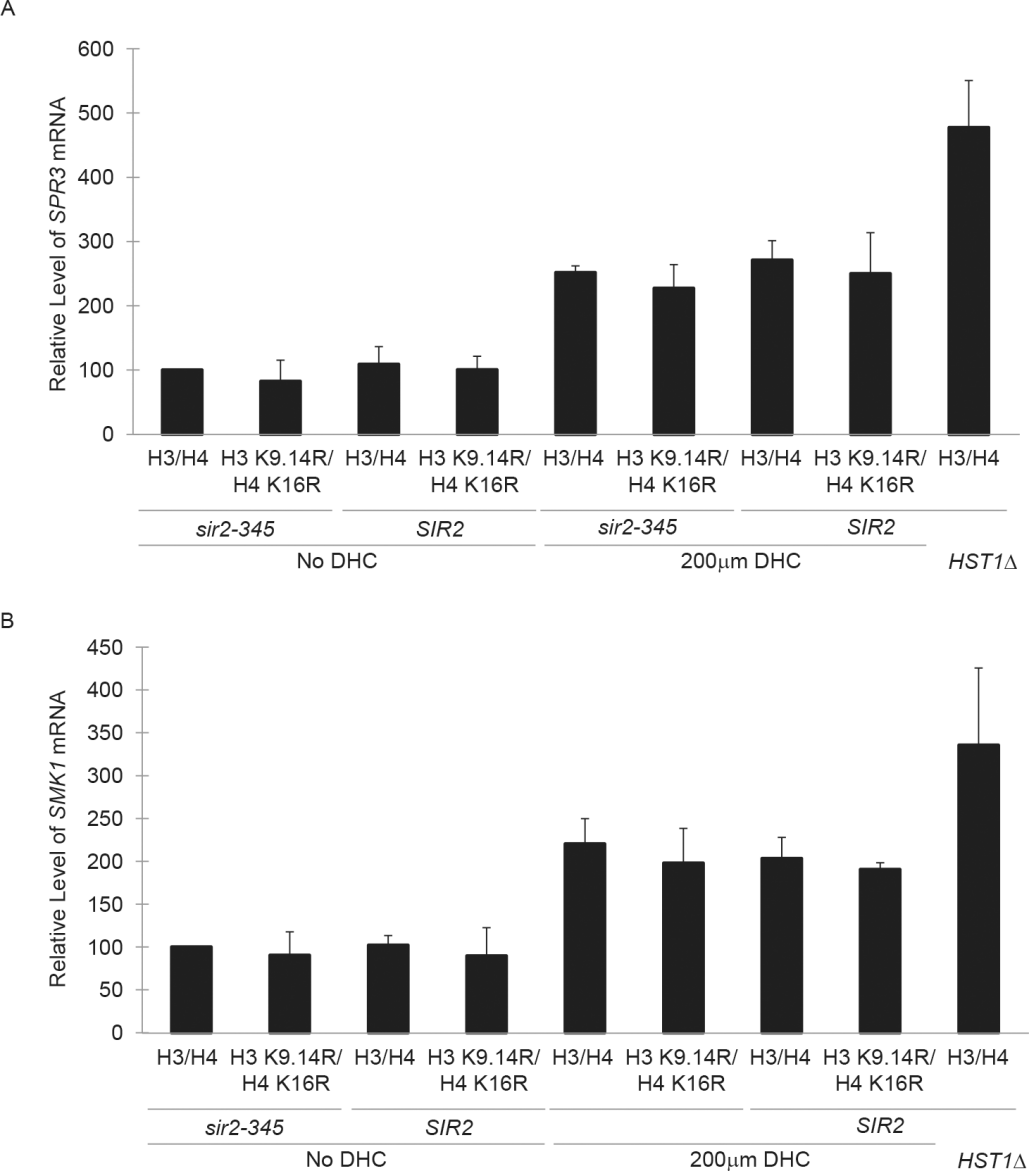
DHC disrupts Hst1p-dependent repression of midsporulation genes. Transcript levels of *SMK1* (**a**) or *SPR3* (**b**) relative to *SCR1* were determined by qRT-PCR for *SIR2* or *sir2-345* strains expressing wild-type or hypoacetylated H3/H4 mutants in the presence or absence of DHC, and compared to *sir2-345* expressing wild-type H3/H4 in the absence of DHC, which was set to 100%. Data was calculated as: 2[(LocusCT − *SCR1*CT)*SIR2* − (LocusCT − *SCR1*CT)*sir2-345* H3/H4] (AVG ± SD, n = 3).

Whether yeast Sirtuins in addition to Sir2p are sensitive to DHC has not been examined previously. Therefore, to assess the specificity of DHC, we next evaluated whether DHC could also target other NAD^+^-dependent histone deacetylases in yeast by assessing the effects of DHC on transcription of two mid-sporulation genes, *SPR3* and *SMK1* and on acetylation of H3 K56 (Fig 5 and S1 Fig, respectively). *SPR3* and *SMK1* are normally maintained in a transcriptionally repressed state during mitotic cell cycles by the deacetylase Hst1p, but become reactivated in *hst1∆* mutants relative to wild-type cells (Fig 5) [20]. Similar to cells lacking *HST1*, in the presence of DHC, *SPR3* and *SMK1* became partially derepressed in logarithmically growing cell cultures as measured by quantitative real-time PCR analyses of mRNA transcripts, implying that DHC inhibited Hst1p in addition to Sir2p.

The NAD^+^-dependent histone deacetylases encoded by *HST3* and *HST4* deacetylate H3 K56ac [22,88,89], a modification important for promoting nucleosome assembly and remodeling, telomeric silencing and responses to DNA damage [23,72,89–96]. To assess the impact of DHC on Hst3p/Hst4p-dependent histone deacetylation, H3 K56ac levels were monitored in wild-type cells and *sir2-345* mutants in the absence and presence of DHC by protein blot analyses, and H3 K56ac levels did not change dramatically under the conditions tested (S1 Fig). Together, these results as well as those above are consistent with DHC being capable of inhibiting multiple Sirtuin family members to varying degrees (see also [7,80,87]).

### Pharmacokinetics of DHC and MA in plasma and interstitial fluid

**Fig 6:**
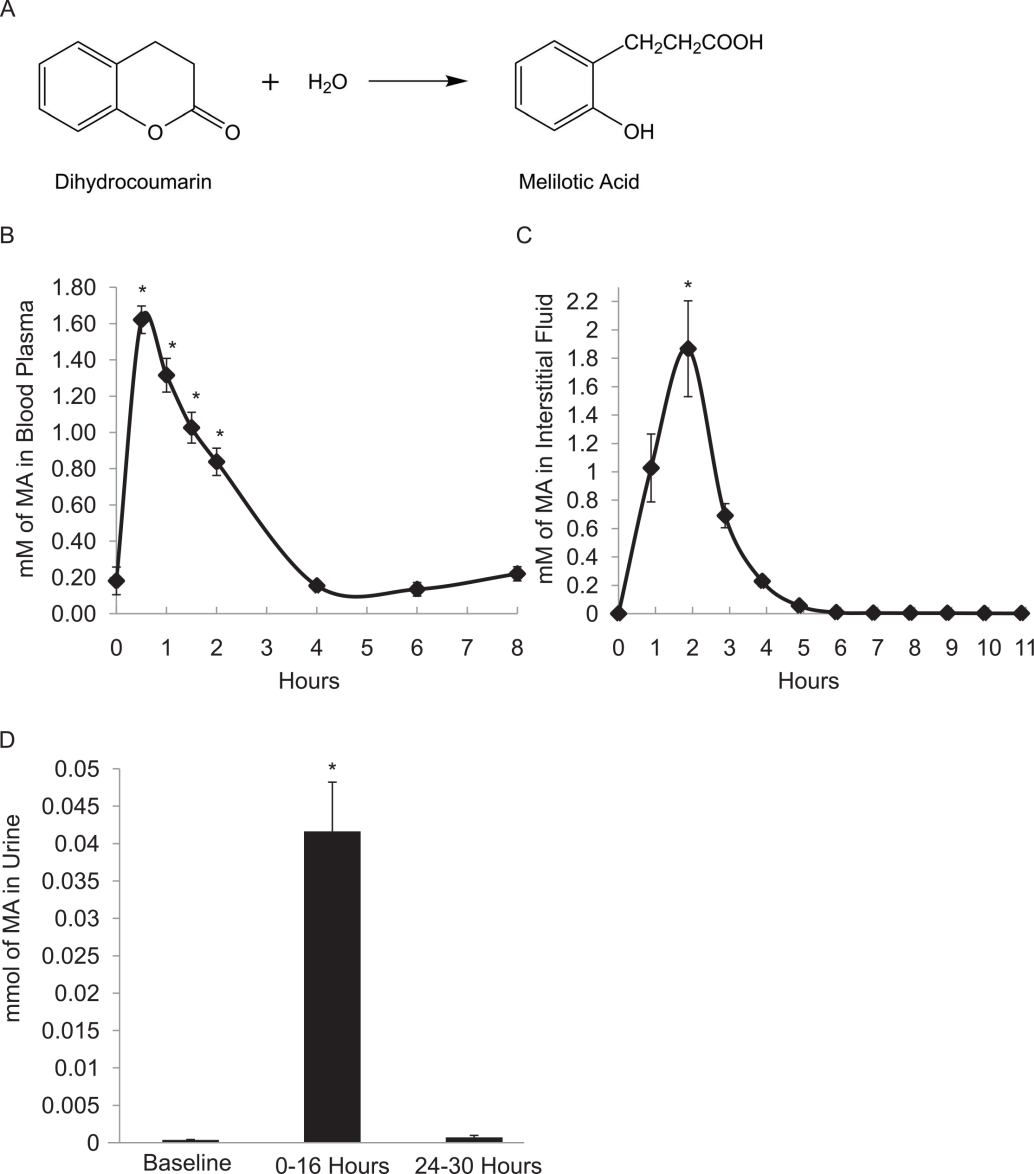
Pharmacokinetics of MA in blood plasma, interstitial fluid, and amount of MA in urine. **a** Scheme of conversion of DHC to MA. **b** MA in the plasma (AVG ± SD, n = 12) was automatically collected from the animals, centrifuged to remove red blood cells, and stored at -80°C until extraction and HPLC analysis as outlined in Materials and methods. **c** The interstitial fluid (AVG ± SE, n = 12) was also automatically collected from the animals and stored at -80°C until direct analysis (without extraction) by HPLC. **d** Urine (AVG ± SE, n = 11) was collected from the rats before gavage, 0–16 hr after gavage, and 24–30 hr after gavage. Particulates were removed from the urine by centrifugation and stored at -80°C prior to extraction and analysis as in Fig 7. * Indicates a significant difference relative to other time points for all experiments.

Humans are exposed to the botanical and dietary bioactive compound DHC in their diets with unknown potential epigenetic consequences. To assess the potential for a dietary impact of DHC, we evaluated the pharmacokinetics, tissue distribution and accumulation of DHC upon ingestion using a Sprauge-Dawley rat model. To investigate the fate of DHC upon ingestion, DHC was administered to the animals at 100 mg/kg body mass by gavage (see Materials and methods) and DHC and related metabolites were evaluated in the blood plasma and interstitial fluid hourly for twelve hours post-gavage. Interestingly, DHC was not detected in the plasma or interstitial fluid after dosing the animals. Instead, a major predicted metabolite of DHC [10], melilotic acid (MA) (Fig 6A) was present. *In vivo*, MA in the plasma peaked at 0.5 hr, consistent with rapid gastric absorption (see below). MA in the plasma then decreased, exhibiting a half-life of 1.7 hr (p < 0.01). After 4 hr, the level of MA in the plasma was statistically at baseline, indicating MA had been cleared from the circulatory system (Fig 6B). Consistent with MA progressing through the circulatory system and into interstitial fluid, MA in the interstitial fluid peaked 2 hr after dosing (p < 0.0001 when compared to all other time points), and was then cleared by 5 hr post-gavage (Fig 6C). As accumulation of DHC in the plasma had not been observed, we next evaluated the stability of DHC when added directly to plasma to yield a 1.66 mM solution *in vitro* (similar to the HPLC analysis outlined below; see also Materials and methods). After ~5 min of incubation, 0.297 ± 0.021 mM MA was observed, and DHC was no longer detected, implying factors present in serum promoted rapid conversion of DHC to MA and/or other metabolites. Our identification of MA as the major metabolite in plasma and interstitial fluid was consistent with capacity of DHC and other lactone ring compounds such as the Sirtuin inhibitor splitomicin to hydrolyze to open structures [10,87] (see Fig 6A, below and Discussion).

### DHC and MA are present in tissues and the digestive tract of rat

**Fig 7:**
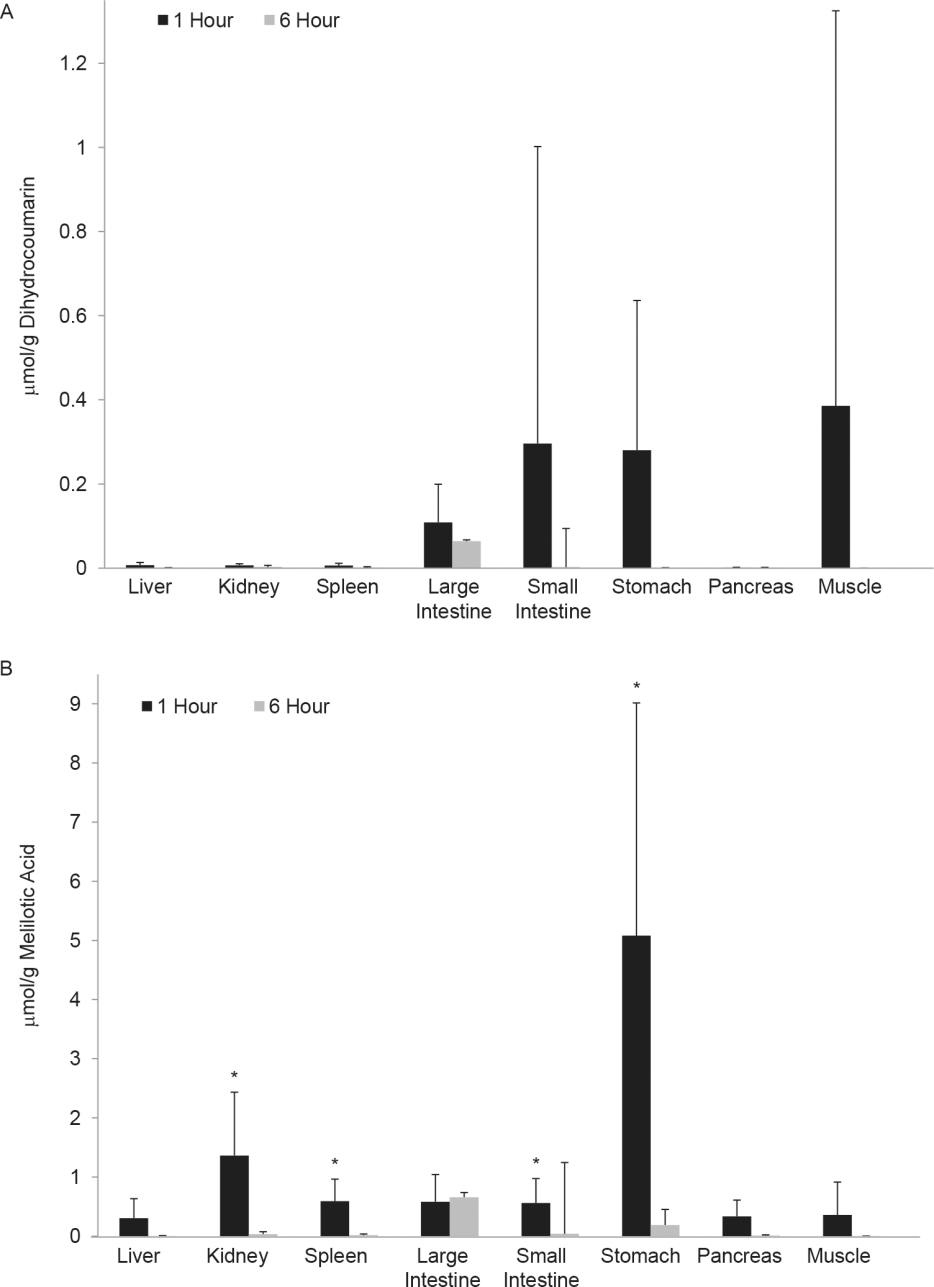
DHC and MA accumulate in digestive-tract tissues. For *in vivo* bioavailability analyses, rats were given 100 mg/kg DHC by gavage. DHC (**a**) and MA (**b**) were extracted from flash frozen tissues using ethyl acetate, dried, resuspended in 50% methanol, and analyzed by HPLC. The amount (μmol) of MA or DHC was quantified by determining the area under the peaks and compared to a standard curve. *Significantly more MA (**b**) was present at 1 hr relative to 6 hr in stomach, small intestine, spleen, and kidney, p = 0.028, 0.027, 0.026, and 0.029, respectively. AVG ± SE, n = 6. See also S3 Fig.

Forty eight hours after their initial dose, the same rats used to evaluate the pharmacokinetics of DHC above were then fed a second dose of 100 mg DHC/kg body mass by gavage and the distribution and tissue accumulation of DHC were evaluated at 1 and 6 hr after dosing. Both DHC and MA were observed in the digestive tract tissues and more MA than DHC was observed in the tested tissues (Fig 7, S3 Fig). More MA was present in the stomach (p < 0.03), small intestine (p < 0.03), spleen (p < 0.03), and kidney (p < 0.03) at 1 hr relative to 6 hr after dosing, and DHC and MA were mostly cleared from the animals by 6 hr after dosing. However, some MA remained in the large intestine tissue at 6 hr after dosing (Fig 7B, S3 Fig).

**Fig 8:**
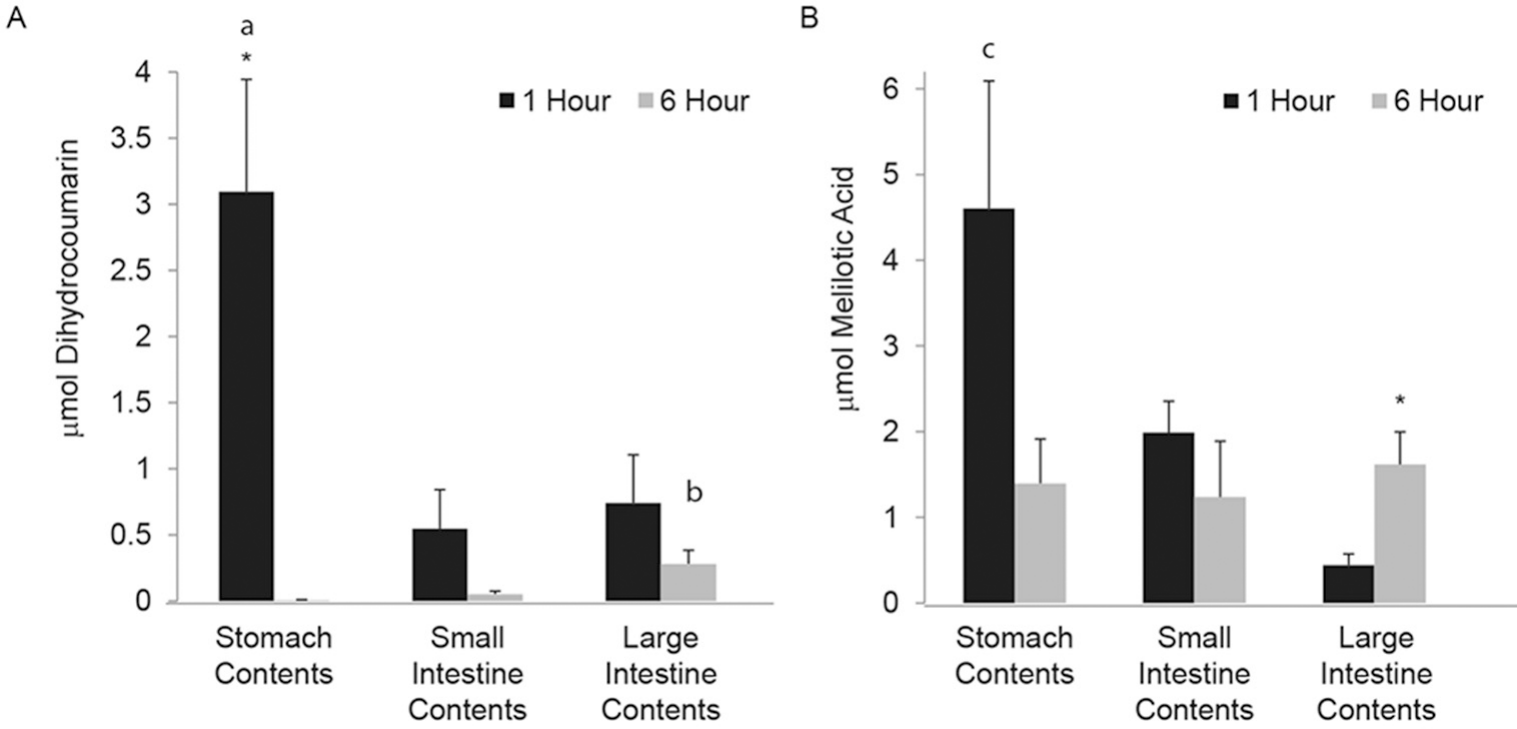
DHC and MA are present in intestinal contents. DHC (**a**) and MA (**b**) were extracted from the contents of the stomach, large intestine, and small intestine, and analyzed by HPLC as outlined Materials and methods. *More DHC was present in the stomach contents at 1 hr vs. 6 hr (p = 0.015) and more MA was present in the large intestine contents at 6 hr vs. 1 hr (p = 0.03); **a** More DHC was present in stomach contents at 1 hr compared to large and small intestine contents at 1 hour (p = 0.0097); **b** more DHC was present in large intestine contents at 6 hr compared to stomach and small intestine contents at 6 hr (p = 0.014); and **c** more MA in was present in stomach contents at 1 hr compared to large intestine contents at 1 hr (p = 0.015). AVG ± SE, n = 6.

Both DHC and MA were also detected in the contents of the digestive tract (stomach, small intestine, and large intestine) (Fig 8). The relative amount of DHC in the stomach contents at 1 hr after dosing was greater than at 6 hr (p < 0.02) and DHC in the stomach contents at 1 hr was also greater than in the small and large intestine contents at 1 hr (p < 0.01). Consistent with progression of the dietary factor though the digestive tract, DHC levels in the large intestine contents 6 hr after dosing were greater than those in the stomach and small intestine at 6 hr (p < 0.02). Nearly all detectible DHC in the digestive tract was cleared by 6 hr (Figs 7A and 8A, see S3 Fig for GI tissue data) and more MA than DHC was observed in the digestive contents at both 1 and 6 hr (Fig 8), implying that DHC had begun to break down within the digestive tract prior to absorption. Less MA was present in all other tissues and contents at 6 hr relative to 1 hr post-gavage. Consistent with DHC and MA being cleared from most tissues by 6 hr, the majority of MA was detected in the urine 0–16 hr after dosing, and very little MA accumulated 24–30 hr after dosing (Fig 6C). Thus, most, if not all, DHC was metabolized to MA and cleared from the animals within 24 hr of ingestion.

### DHC and MA are unstable during digestion

**Fig 9:**
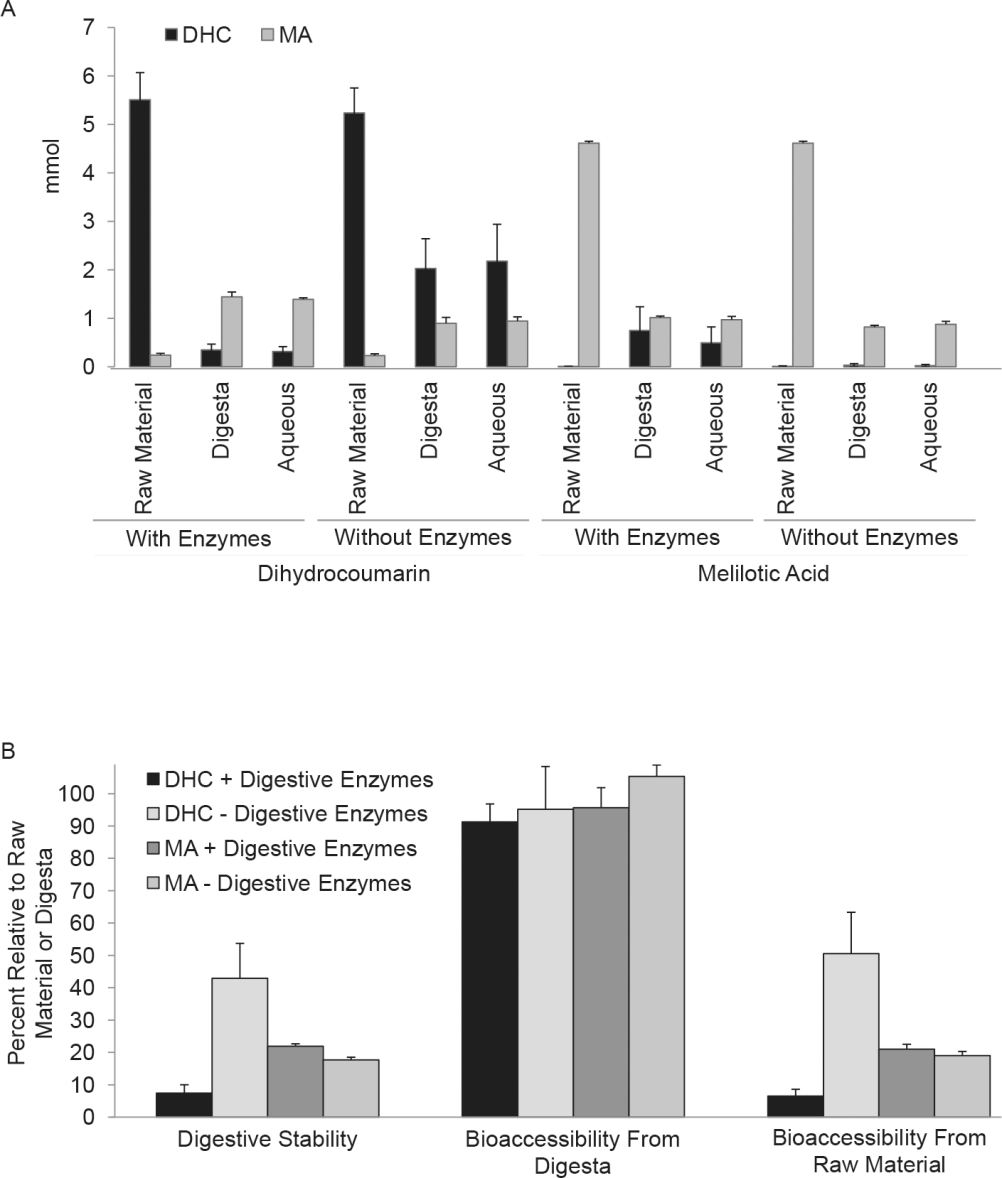
DHC and MA are unstable through digestion. **a** Amount (mmol) of DHC or MA through stages of digestive simulation (see Materials and methods). Aliquots from the initial sample (raw material), gastric phase (digesta), and after micellar fractionation (aqueous) were extracted and analyzed by HPLC as outlined in Materials and methods. **b** Digestive stability and bioaccessibility were calculated for DHC (AVG ± SE, n = 7) and MA (AVG ± SE, n = 6). Digestive stability = digesta /raw material; Bioaccessibility from digesta = aqueous/digesta; Bioaccessibility from raw material = aqueous/raw material.

The above results implied DHC localized to the digestive tract of the rat (Figs 7 and 8) and was rapidly converted to MA, potentially prior to its being absorbed by digestive tissues. Therefore, to assess the stability of DHC and MA during typical human digestion, an *in vitro* simulation of the digestion process in the human stomach and small intestine was performed (Fig 9). In this assay, DHC or MA was exposed to human digestive conditions simulated by a static model using porcine enzymes, average pH conditions for gastric and small intestinal conditions, and incubation at physiological conditions, 37°C. In this assay, samples collected after the gastric plus small intestine phase are referred to as the digesta and fractions collected after micellarization are referred to as the aqueous phase. When DHC was the starting material in the digestive simulation in the presence of digestive enzymes, equal amounts of DHC were observed in the digesta and in the aqueous phase. Similarly, when MA was the starting material in the presence of digestive enzymes, similar amounts of MA were observed in the digesta and in the aqueous phase (Fig 9A; see also Bioaccessibility from Digesta in Fig 9B). These results indicated that breakdown of DHC or MA occurred primarily during the gastric and small intestine phase of digestion. The remaining DHC or MA was stable upon transfer to bile salt lipid micelles as the amount of DHC or MA present in the digesta relative to aqueous fraction was >90% (see Bioaccessibility from Digesta in Fig 9B). Approximately 7% of DHC and 20% of MA remained bioaccessible after both digestion and micellarization (see Bioaccessibility from Raw Material in Fig 9B). Thus, despite its limited digestive stability, some DHC, as well as MA, remained modestly available for absorption after digestion (see below).

To assess the effect of pH on the stability of DHC or MA, control experiments were also conducted in the absence of digestive enzymes and DHC was still converted to MA, albeit less efficiency. Changes in pH reduced the bioavailability of DHC to ~50% and MA to ~18% of the input amounts (Fig 9A and 9B). Together, these results indicated both changes in pH and exposure to digestive enzymes contributed to the breakdown of DHC in the digestive tract. Surprisingly, DHC accumulated in samples in which the starting material had been MA (Fig 9A), implying that either MA converted to DHC spontaneously or the digestive enzymes promoted the conversion of MA into DHC. To assess whether digestive enzymes facilitated the conversion of MA to DHC, an *in vitro* digestion experiment was conducted under conditions in which the digestive enzymes were inactivated by heating to 100°C prior to their use in the assay. Under these conditions, DHC was still detected by HPLC, implying that the conversion of MA to DHC could occur enzyme-independently (data not shown).

### DHC converts to MA in aqueous environments

**Fig 10:**
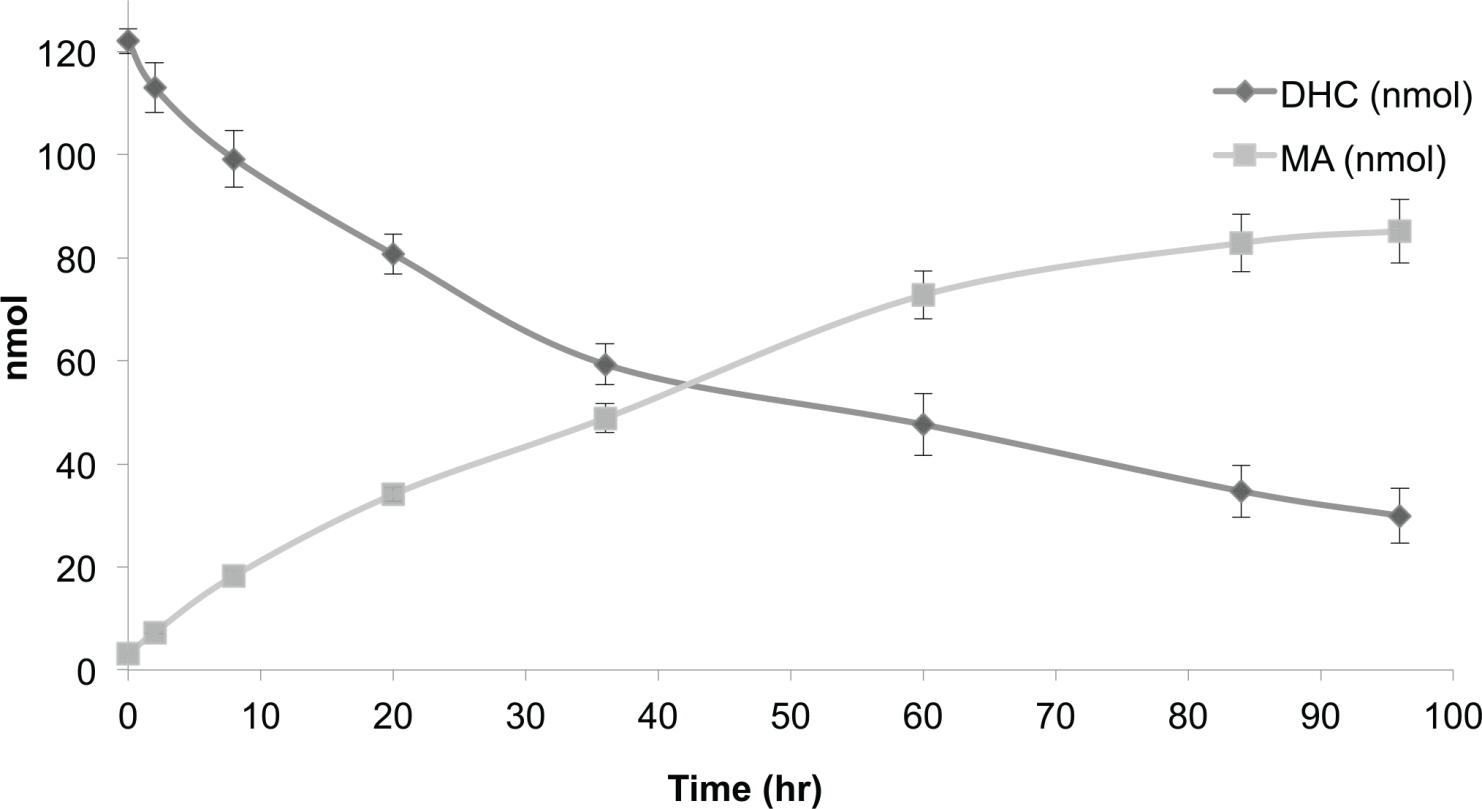
DHC converts to MA in aqueous environments. DHC was dissolved in rich (YPD) medium and amount of DHC and MA present as a function of time was determined by HPLC and quantified using standard curves (AVG ± SD, n = 3).

**Fig 11:**
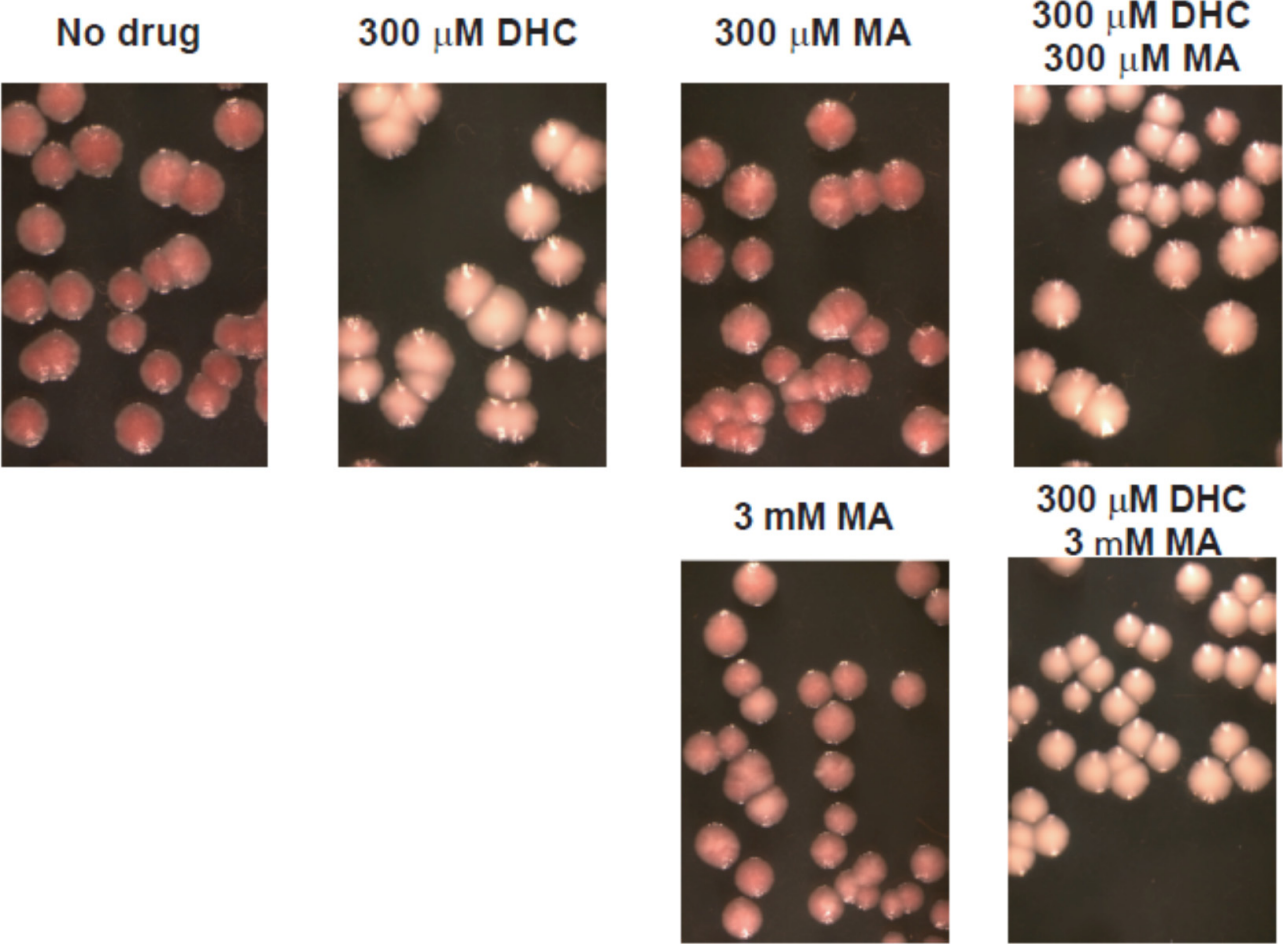
MA disrupts silencing of *MAT*::*ADE2* less efficiently than DHC. Logarithmically growing cells were plated on rich (YPD) media containing the indicated concentrations of MA and/or DHC, and analyzed as outlined in the Materials and methods.

The above experiments indicated that DHC was unstable and had been converted to MA both *in vivo* (Figs 6, 7 and 8) and *in vitro* (Fig 9). This raised the possibility that the effect on Sirtuin-dependent phenotypes attributed to DHC in Fig 1 could instead, or additionally, be mediated by MA. To assess the stability of DHC in the aqueous environment used to assess its impact on Sir2p-dependent silencing, DHC was incubated in rich medium (YPD) and monitored over 96 hr by HPLC analysis. After 36, 60, and 96 hr, 49%, 39%, and 26%, respectively, of DHC remained present in YPD, indicating that DHC was unstable in aqueous environments and converted to MA (Fig 10). Furthermore, these results implied that less DHC than noted in Fig 1 and Fig 11 was likely sufficient to disrupt silencing as the plates for these experiments had been poured ~24 hr prior to use and yeast had been incubated on the plates two to five days prior to collecting data. Therefore, maximally, ~25% of DHC would have been expected to remain in the media and ~75% would have been converted to MA by the completion of these experiments. In other words, this finding raised the possibilities that MA was a bioactive compound, exposure to less DHC than originally anticipated was sufficient to disrupt silent chromatin or both DHC and MA could compromise silencing. To compare the bioactivity of DHC or MA, we repeated the experiment shown in Fig 1 and monitored silencing *HMR*::*ADE2* in the presence of DHC, MA or DHC plus MA. Roughly 30–100 fold more MA than DHC was required to achieve similar defects in the maintenance of silent chromatin (compare Fig 1 to Fig 11) and these silencing defects were exacerbated in the presence of DHC plus MA (Fig 11). Therefore, the major bioactive compound responsible for disrupting Sir2-dependent processes in the experiments shown in Figs 1–5 was likely DHC. Whether the MA-dependent effects are due to direct inhibition of Sir2p or conversion of a portion of the MA back into DHC in the media or after being taken up by yeast is unknown.

ABC transporters have previously been implicated in preventing the accumulation of plant polyphenols to bioactive levels in mammalian cells [97]. To assess cellular uptake of DHC and the impact of DHC on silencing within a time frame in which DHC levels were expected to remain relatively stable and under conditions in which we hypothesized intracellular levels of DHC would accumulate, transient α-factor confrontation assays were conducted in wild-type yeast and *pdr5* mutants. *pdr5* mutants lack a major ABC transporter that confers natural multi-drug resistance to yeast [98,99]. In α factor confrontation assays, *MAT****a*** yeast arrest in G1 and adopt a ‘shmoo’ morphology in the presence of the α-factor pheromone. However, if Sir2p-dependent silencing is lost at the silent mating-type locus *HML* in *MAT****a*** yeast, simultaneous expression of both ***a*** and α mating information will occur and cells will fail to maintain an arrest in G1. Logarithmically growing strains were treated with α-factor alone or α-factor plus DHC, and incubated for 3, 4 or 5 hr, then cells were fixed in ethanol. Cell morphologies were scored for 100 cells for each time point and treatment. Wild-type strains treated with DHC broke out of the α-factor arrest and began cycling after 4 hr in 500 μM DHC. In contrast, *pdr5*∆ mutants began to break out of the arrest in only 100 μM DHC (Table D in S1 File). Together, these results implied that DHC was likely the major bioactive compound disrupting Sirtuin function. These results also indicated that the multi-drug transporter Pdr5p conferred resistance to DHC, and were consistent with the intracellular concentration of DHC required to disrupt Sir2p function being lower than had been initially estimated by the amount of DHC used in colony color and growth assays (Fig 1, Fig 11).

## Discussion

Dietary components have the potential to affect epigenetic processes and thereby influence expression of genes that promote health or disease. Using budding yeast as a model, this study demonstrated that the natural plant phenolic and commercial dietary factor DHC adversely affected numerous NAD^+^-dependent deacetylase-regulated processes. Results reported here demonstrated, for the first time, exposure to DHC mechanistically led to defects in both establishing and maintaining Sir2p-dependent silent chromatin, and disrupted Sir protein propagation along the chromosome (Figs 1, 2, 3, 4, 11 and S2 and Table D in S1 File). Exposure to DHC also caused defects in Hst1p-dependent repression of meiosis-specific genes during the mitotic cell cycle (Fig 5), but did not dramatically alter Hst3p/Hst4p-dependent regulation of H3 K56ac (S1 Fig), consistent with DHC being a selective inhibitor of Sirtuins that overlaps in specificity with splitomycin [80,87,100]. We also provide novel evidence that MA causes silencing defects, albeit less efficiently than DHC (Fig 11). In addition, we demonstrate ABC transporters can counteract the accumulation of DHC to bioactive levels in cells (Table D in S1 File). Previously, DHC has been shown to inhibit the mammalian Sirtuin orthologs SIRT1 and SIRT2 in addition to Sir2p and to affect Sirtuin-regulated acetylation of p53 and apoptosis *in vitro* [7,87]. Together, these results extend the current knowledge of the modes of action of DHC and imply that DHC is a broad-acting inhibitor of Sirtuins that has the potential to disrupt processes regulated by a subset of Sirtuin family members.

The impact of dietary components on biological processes upon ingestion is affected by their digestive release and stability, intestinal absorption, metabolic processing, distribution, and rate of clearance from the body. Here, we applied a combination of *in vitro* and rat models to conduct a detailed assessment of the pharmacokinetic parameters of DHC. Collectively, the bioavailability data implied that upon ingestion, DHC was readily hydrolyzed to MA and rapidly absorbed (Fig 6A). The proportion of DHC that accumulated in tissues reflected only a small fraction of the ingested sample (Fig 7). Instead, DHC was efficiently converted to MA, a less bioactive form, in the plasma under conditions simulating passage through the digestive tract, and in aqueous environments (Figs 9 and 10 and data not shown). Consistent with these findings, DHC as well as MA were detected within the digestive tract contents (Fig 8) and both DHC and MA were rapidly observed in tissues (Fig 7, S3 Fig). However, as only MA was detected in interstitial fluid, plasma, and urine, our findings are consistent with the majority of the remaining DHC being efficiently converted to MA upon absorption and MA being readily cleared from the body (Fig 6). Under the conditions tested, neither MA nor DHC accumulated to levels anticipated to be capable of broadly disrupting Sirtuin-dependent processes *in vivo*, reducing potential concern for unanticipated impacts of DHC as a dietary compound on Sirtuin-mediated functions in healthy individuals. These bioavailability profiles for DHC have similarities to those observed for anthocyanins, carotenoids and tocochromanols and the pharmacokinetics of MA in plasma (Fig 6A) is similar to that of anthocyanins [101–104] (see also [105]).

The instability of DHC in aqueous solutions (Fig 10), at low pH (Fig 9), and in the presence of digestive enzymes (Fig 9) may have contributed to the variable levels of DHC observed between rats for most tissues (Fig 7). The low levels of DHC in tissues and the absence of DHC in the plasma, interstitial fluid, and urine may also be related to uptake of DHC from the gut (see below) or the conversion of DHC to MA or other compounds via enzymes in the gut, tissues and bloodstream. In plants that actively synthesize dihydrocoumarin such as *Melilotus alba*, dihydrocoumarin is converted to melilotate by a dihydrocoumarin hydrolase, but this has not been identified in mammals [6]. In mammals, one possible route for the metabolism of DHC upon absorption may involve PON glycoproteins encoded by the human paroxonase gene family, PON1, PON2 and PON3 [106–111]. PON1 hydrolyzes aliphatic lactones, and DHC is commonly used as a substrate for all three PON enzymes [112]. PON1 is synthesized in the liver, secreted into the blood stream and associates primarily with high density lipoproteins [106–108]. PON2 is expressed in brain, liver, kidney, and testes [109,110], whereas PON3 is expressed primarily in the liver and the kidney [111]. Whether the bioavailability of DHC or similar compounds are altered in individuals with reduced PON activity, such as those with certain PON1 polymorphisms or varied PON expression levels, chronic alcoholic liver disease or acute Hepatitis B viral infections [112–114], relative to the general population is unknown.

Another candidate for affecting the bioavailability of DHC was raised from our observation that loss of the ABC transporter Pdr5p conferred resistance to DHC in yeast (Table D in S1 File). Homologous mammalian multi-drug transporters would be predicted to influence absorption and accumulation of DHC or MA in rat tissues similarly (Fig 7). Consistent with this model, ABC transporters/P glycoproteins are well known to negatively affect the bioavailability of numerous drugs by counteracting their uptake in the intestinal epithelia or by influencing their metabolism [115,116]. Likewise, overexpression of an ABC transporter has previously been implicated in resistance of tumor cells to the plant polyphenol curcumin in culture [97].

The results reported here reflect those obtained upon delivery of DHC combined with water and a surfactant. The bioavailability of botanicals/plant secondary metabolites as well as their stability or conversion of dietary components to their most bioactive forms can be influenced significantly by food composition and formulations [102,117–120] as well as other factors including microbes in the digestive tract [105,121–123], which e.g. can metabolize coumarin into DHC and MA [124]. Such factors have the potential to influence the digestive stability and uptake of dietary components or conversion to bioactive forms and therefore, the efficacy of the consumed compounds or their subsequent metabolites. These will also eventually need to be considered for a complete understanding of the potential for impact of environmental and dietary factors on epigenetic processes.

Our bioavailability and pharmacokinetic data extend findings from limited animal studies evaluating the metabolism of DHC, which itself is generated during the metabolism of coumarin [14], that were performed largely in the 1950’s and 1960’s prior to the discovery of Sir proteins. These studies administered DHC, MA, and other coumarin derivatives primarily to rats, hamsters, or rabbits, focused on assessing the toxicity of coumarins and provided a limited analyses of the tissue distribution of coumarin derivatives [11–14,125]. Other studies on the metabolism of coumarin, 7-hydroxycoumarin, and MA in rats, mice, or Syrian hamsters [126–128] also did not evaluate accumulation or distribution of these coumarin derivatives in tissues but, consistent with our observations, did report clearance of coumarin derivatives via urine. DHC has previously been assessed for toxicity and carcinogenicity mouse and rat models, but pharmacokinetics and the molecular basis for observed effects were not explored in these studies [13,129]. Our demonstration that DHC is efficiently converted to MA upon ingestion implies that findings attributed to DHC in these studies likely also, or alternatively, have been the result of exposure to MA.

Together, our findings imply that at average daily consumption levels by humans [10], DHC and its metabolite MA are unlikely to accumulate in tissues to levels that would result in wide-spread acute disruption of Sirtuin-dependent processes. However, levels of dietary phenolics that promote or prevent disease can vary greatly and evidence is growing that dietary and environmental factors can have long-term effects on gene expression via influencing epigenetic processes [130]. Either transient or chronic dietary exposure to DHC or other modulators of Sirtuin function have the potential to result in occasional de-repression events at individual Sirtuin-regulated loci that could lead to stably inherited changes in gene expression states. This scenario would be analogous to low probability loss-of-silencing events observed at the *ADE2* and *TRP1* reporter genes at *HMR* in individual yeast cells that then gave rise to sectors of stable de-repressed cell populations or to colonies capable of growing in the absence of tryptophan (Figs 1 and 11). However, as DHC did not accumulate in tissues under the conditions tested, such events would be predicted to be rare, but the frequency of such events could be increased in sensitized backgrounds via interactions with other environmental, dietary or genetic factors, particularly at sites of higher exposure such as the epithelium of the digestive tract. Dietary exposure to inhibitors of Sirtuin function such as DHC also has the potential to promote health. Enterocyte-specific inactivation of SIRT1 reduces the overall intestinal tumor load in the APC^+/min^ mouse model for colon cancer [131]. As tumor size, rather than tumor frequency is decreased in this genetic background, SIRT1 likely influences post-initiation events in this system.

Numerous natural compounds linked to health are capable of influencing histone and/or DNA modifications, and therefore also have the potential to modulate epigenetic processes, including lycopene (tomato), phloretin (apple), hesperidin (citrus), naringenin (citrus), and protocatechuric acid (olives), anacardic acid (cashews), allyl mercaptan (garlic), isoliquiritigenin (liquorice), curcumin, genistein (soybean), caffeic acid (coffee), coumaric acid (cinnamon), catechins (tea), isothiocyanates (broccoli), and picetannol (blueberries; grapes) [1,132]. How DHC and such other micronutrients interact at the dietary level for chemoprevention or -promotion has only begun to be explored. Combined exposures to bioactive phytochemicals, including mixtures of polyphenols or polyphenols plus other kinds of phytochemicals, as well as phytochemicals with dietary micronutrients or drugs, can lead to suppressive or synergistic interactions [133]. However, controlled chronic dosing regimens that mimic dietary exposures will likely be required to clarify the bioaccumulation and long-term impacts on health of daily ingestion of DHC and other bioactive dietary components that influence epigenetic processes.

In summary, compelling evidence is accumulating that plant secondary metabolites found in foods or their derived ingredients including additives like DHC have the ability to modulate epigenetic processes by impacting the activities of Sirtuins or other enzymes regulating gene silencing or activation. What remains to be elucidated is how and the frequency with which low-level dietary or environmental exposures induce heritable somatic or germ-line changes in gene expression states to clarify how such exposures can directly contribute to phenotypes associated with health or disease.

## Supporting Information

S1 Fig H3 K56ac levels are not affected by the presence of DHC. Whole cell extracts of *SIR2* or *sir2-345* cells expressing wild-type or hypoacetylated H3/H4 grown logarithmically in the presence or absence of DHC were analyzed by immunoblots using anti-H3 K56ac and anti-H3 antibodies as a loading control.
(TIF)

S2 Fig DHC prevents Sir spreading. **a** Sir2p, **b** Sir3p and **c** Sir4p binding at *MAT*, *HMR-E*, ***a1***, and *HMR*-*I* in the presence of 200 μm DHC in *SIR2* or *sir2-345* strains expressing wild-type or hypoacetylated H3/H4 mutants were monitored by ChIP using IgG, anti-Sir2p and anti-Sir4p antibodies and qRT-PCR. The efficiency of co-precipitation of each locus is expressed relative to *MAT* and was calculated as described in Fig 3B; Independent replicate of analyses in Figs 3B and 4; see also [85].
(EPS)

S3 Fig DHC and MA accumulate in digestive-tract tissues. Rat *in vivo* bioavailability data for MA or DHC from Fig 7, is displayed as the amount (μmol) of MA or DHC present at 1 hr or 6 hr per gram of total GI organ tissue (stomach, small intestine, plus large intestine for each animal) to control for ad libitum feeding, plus animal-to-animal variation in GI mobility and digestion. AVG ± SE, n = 6. More MA than DHC was present in GI tissues at 1 hr (or 6 hr), p = 0.076 (or p = 0.0011), and more MA (or DHC) was present in GI tissues at 1 hr vs. 6 hr, p = 0.032 (or p = 0.021); Wilcoxon rank sum test).
(EPS)

S1 File Supplementary Tables. Yeast Strains Used in This Study (**Table A**), Plasmids Used in This Study (**Table B**). Oligonucleotides Used in This Study (**Table C**). Loss of the Multidrug Transporter *PDR5* Increases Sensitivity of Yeast to DHC in α-Factor Confrontation Assays (**Table D**).
(PDF)

## Abbreviations

DHC: dihydrocoumarin
MA: melilotic acid
