# Supplementary figures and images for: Impacts on Sirtuin Function and Bioavailability of the Dietary Bioactive Compound Dihydrocoumarin
Source: PLoS One. 2016 Feb 16;11(2):e0149207. doi: 10.1371/journal.pone.0149207 (PMC4755582; doi:10.1371/journal.pone.0149207)

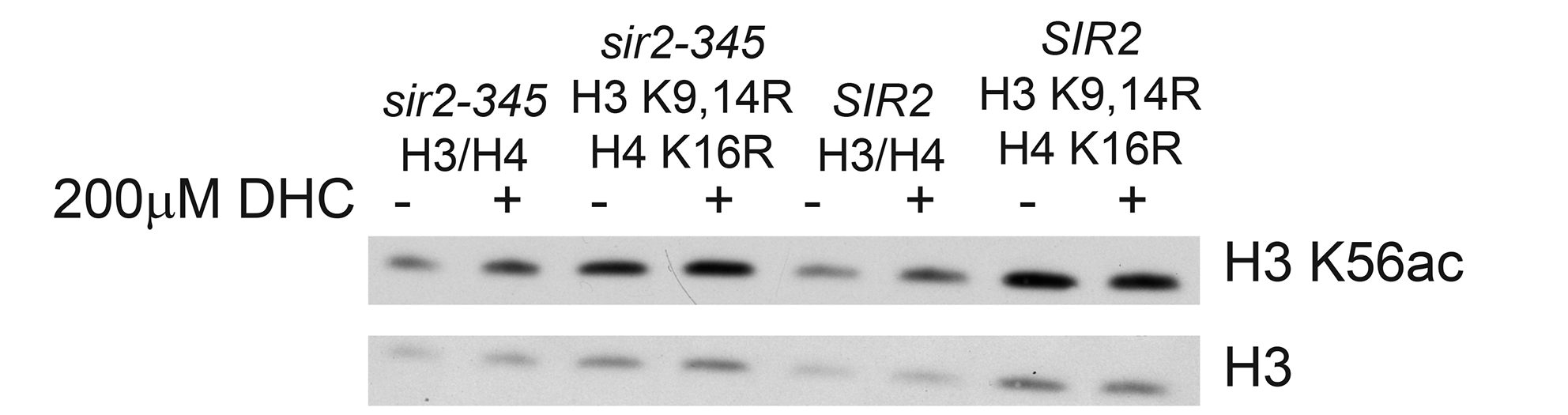

Supplement: S1 Fig — Whole cell extracts of SIR2 or sir2-345 cells expressing wild-type or hypoacetylated H3/H4 grown logarithmically in the presence or absence of DHC were analyzed by immunoblots using anti-H3 K56ac and anti-H3 antibodies as a loading control. (TIF) [file pone.0149207.s001.tif]
