# Supplementary material for: Impacts on Sirtuin Function and Bioavailability of the Dietary Bioactive Compound Dihydrocoumarin
Source: PLoS One. 2016 Feb 16;11(2):e0149207. doi: 10.1371/journal.pone.0149207 (PMC4755582; doi:10.1371/journal.pone.0149207)
Supplement: S1 File — Yeast Strains Used in This Study (Table A), Plasmids Used in This Study (Table B). Oligonucleotides Used in This Study (Table C). Loss of the Multidrug Transporter PDR5 Increases Sensitivity of Yeast to DHC in α-Factor Confrontation Assays (Table D). (PDF) [file pone.0149207.s004.pdf]

Supplementary Information

Table A. Yeast Strains Used in This Study.

Yeast	Genotype	Source
W303	*MAT**a*** or *α ade2-1 his3-11,15 leu2-3,112 trp1-1 ura3-1 can1-100*	R. Rothstein
JRY3350	W303 *MAT**a** HMR::TRP1 hht1-hhf1Δ::LEU2 hht2-hhf2Δ::HIS3* plus pMP3	J. Rine
AKY2101	W303 *MAT**a** HMR::ADE2 hht1-hhf1Δ::LEU2 hht2-hhf2Δ::HIS3* plus pMP3	[1]
CBY37	W303 *MAT**a** bar1⁻*	C. Beh
BY4741	*MAT**a** his3Δ1 leu2Δ0 met15Δ0 ura3Δ0*	[2]
AKY5047	BY4741 *pdr5Δ::KanMX*	[2]
JRY8012	S288C *MAT**a** his3Δ1 leu2 ura3 met15 pdr5Δ::KanMX snq2Δ::KanMX yor1Δ::KanMX*	[3]
AKY1101[a]	W303 *MATα leu2-3,112::LEU2-sir2-345 sir2Δ::KanMX hht1-hhf1Δ::LEU2 hht2-hhf2Δ::HIS3* plus PK189	[4]
AKY1968[a]	W303 *MATα hht1-hhf1Δ::LEU2 hht2-hhf2Δ::HIS3* plus PK189	[4]
JRY7145	W303 *MATα hst1Δ::KanMX*	L. Rusché
AKY1012	W303 *MATα leu2-3,112::LEU2-sir2-345 sir2Δ::KanMX hht1-hhf1Δ::LEU2 hht2-hhf2Δ::HIS3* plus pAK278 and pJR69	This study
AKY3131	W303 *MATα leu2-3,112::LEU2-sir2-345 sir2Δ::KanMX hht1-hhf1Δ::LEU2 hht2-hhf2Δ::HIS3* plus pwz-414-F13 and YCp50	This study
AKY2825	W303 *MATα leu2-3,112::LEU2-sir2-345 sir2Δ::KanMX hht1-hhf1Δ::LEU2 hht2-hhf2Δ::HIS3* plus pAK923 and pJR69	This study
AKY2827	W303 *MATα hht1-hhf1Δ::LEU2 hht2-hhf2Δ::HIS3* plus pAK923 and pJR69	This study

[a]Parental strains used in this study. See Table B in S1 File for descriptions of plasmids that were introduced into AKY1101 and AKY1968 for experiments described in text.

Table B. Plasmids Used in This Study.

Plasmid	Description	Source
pJR69	*SIR2* in YCp50	J. Rine
YCp50	*ARS/CEN/URA3*	[5]
pwz-414-F13	*HHT2 HHF2 ARS/CEN/TRP1*	[6]
PK189	*HHT2 HHF2 ARS/CEN/URA3*	P. Kaufman
pAK 923	H3 K9,14R H4 K16R *ARS/CEN/TRP1*	[4]
pMP3	*HHT2 HHF2 ARS/CEN/TRP1*	[7]

Table C. Oligonucleotides Used in This Study.

Region	Oligonucleotide Pair	Application	Reference
SCR1	oALK402 5’CGCGGCTAGACACGGATT	qRT-PCR	[4]
	oALK403 5’GCACGGTGCGGAATAGAGAA		
HMR E	oALK261 5’CCCGTCCAAGTTATGAGCTTAATCT	ChIP	[4]
	oALK262 5’GGAGTCTTAATTTCCCTGATTTTAGTTTAG		
HMR I	oALK263 5’TTTCTCTCTTCTTTTCCTTTAGTTGGA	ChIP	[4]
	oALK264 5’CATTTAGGAAAAAACGCTAAAGTGTGT		
*HMR**a1***	oALK270 5’TTTAGAAGAAAGCAAAGCCTTAATTCC	ChIP, qRT-PCR	[4]
	oALK271 5’CTTGAAGTGGAGTAATGCCACATT		
MAT	oALK283 5’GCCCCTGGACTACGAAAACTTA	ChIP	[4]
	oALK284 5’ACAATTCATCATTTGCGTTCGTT		
ACT1	oALK648 5'GACGCTCCTCGTGCTGTCTT	qRT-PCR	This Study
	oALK649 5'GTCTTTTTGACCCATACCGACC		
SMK1	oALK670 5'GCAGATGTCATCCACCGAGA	qRT-PCR	This Study
	oALK671 5'CCCTAGCGAGACCAAAATCG		
SPR3	oALK679 5'ACACGGCCAAGACATCGGTA	qRT-PCR	This Study
	oALK680 5'CGCCACCATCAGCGTAAAAT		

Table D. Loss of the Multidrug Transporter *PDR5* Increases Sensitivity of Yeast to DHC in α-Factor Confrontation Assays.

		WT		*pdr5*Δ	
μM DHC treatment	Hours	G1[a]	S/G2/M[b]	G1[a]	S/G2/M[b]
0	4	95	5	95	5
100	4	98	2	82	18
500	4	65	35	54	46
0	5	91	8	82	18
100	5	96	4	61	39
500	5	47	53	48	52

[a]Cells with an unbudded or schmoo morphology.

[b]Cells with budded, large budded, or 'dumbbell' morphology.

Supplementary References

1. Miller A, Yang B, Foster T, Kirchmaier AL (2008) Proliferating cell nuclear antigen and ASF1 modulate silent chromatin in Saccharomyces cerevisiae via lysine 56 on histone H3. Genetics 179: 793-809.

2. Winzeler EA, Shoemaker DD, Astromoff A, Liang H, Anderson K, Andre B, et al. (1999) Functional characterization of the S. cerevisiae genome by gene deletion and parallel analysis. Science 285: 901-906.

3. Jeong H, Herskowitz I, Kroetz DL, Rine J (2007) Function-altering SNPs in the human multidrug transporter gene ABCB1 identified using a Saccharomyces-based assay. PLoS Genet 3: e39.

4. Yang B, Kirchmaier AL (2006) Bypassing the Catalytic Activity of SIR2 for SIR Protein Spreading in S. cerevisiae. Mol Biol Cell 17: 5287-5297.

5. Ma H, Kunes S, Schatz PJ, Botstein D (1987) Plasmid construction by homologous recombination in yeast. Gene 58: 201-216.

6. Zhang W, Bone JR, Edmondson DG, Turner BM, Roth SY (1998) Essential and redundant functions of histone acetylation revealed by mutation of target lysines and loss of the Gcn5p acetyltransferase. EMBO J 17: 3155-3167.

7. Kelly TJ, Qin S, Gottschling DE, Parthun MR (2000) Type B histone acetyltransferase Hat1p participates in telomeric silencing. Mol Cell Biol 20: 7051-7058.
